# Measurement of serum antibodies against NY-ESO-1 by ELISA: A guide for the treatment of specific immunotherapy for patients with advanced colorectal cancer

**DOI:** 10.3892/etm.2014.1913

**Published:** 2014-08-18

**Authors:** YAN-YAN LONG, YU WANG, QIAN-RONG HUANG, GUANG-SHUN ZHENG, SHUN-CHANG JIAO

**Affiliations:** 1 Department of Medical Oncology, Chinese PLA General Hospital, Beijing 100853, P.R. China; 2 Medical School of Nankai University, Tianjin 300071, P.R. China; 3 Beijing ImmunoTech Applied Science Ltd., Beijing 100097, P.R. China

**Keywords:** NY-ESO-1, humoral immunity, serum antibody, enzyme-linked immunosorbent assay, antigen-specific immunotherapy

## Abstract

NY-ESO-1 has been identified as one of the most immunogenic antigens; thus, is a highly attractive target for cancer immunotherapy. The present study analyzed the expression of serum antibodies (Abs) against NY-ESO-1 in patients with advanced colorectal cancer (CRC), with the aim of guiding the treatment of NY-ESO-1-based specific-immunotherapy for these patients. Furthermore, the present study was the first to evaluate the kinetic expression of anti-NY-ESO-1 Abs and investigate the possible influencing factors. A total of 239 serum samples from 155 pathologically confirmed patients with advanced CRC (stages III and IV) were collected. The presence of spontaneous Abs against NY-ESO-1 was analyzed using an enzyme-linked immunosorbent assay (ELISA). The results demonstrated that 24.5% (38/155) of the investigated patients were positive for NY-ESO-1-specific Abs. No statistically significant correlations were identified between the expression of anti-NY-ESO-1 Abs and clinicopathological parameters, including age and gender, location, grading, local infiltration, lymph node status, metastatic status and K-ras mutation status (P>0.05). In 59 patients, the kinetic expression of anti-NY-ESO-1 Abs was analyzed, of which 14 patients were initially positive and 45 patients were initially negative. Notably, 16/59 (27.1%) patients changed their expression status during the study period, and the initially positive patients were more likely to change compared with the initially negative patients (85.7 vs. 8.8%; P<0.001). Therefore, monitoring serum Abs against NY-ESO-1 by ELISA is an easy and feasible method. The high expression rate of NY-ESO-1-specific Abs in CRC patients indicates that measuring the levels of serum Abs against NY-ESO-1 may guide the treatment of NY-ESO-1-based specific immunotherapy for patients with advanced CRC.

## Introduction

Colorectal cancer (CRC) is one of the most common malignancies worldwide. In the USA, despite a small decrease in the incidence and mortality rates during the past two decades, CRC remains the second most common type of cancer (1). Due to distant invasion and migration, the five-year survival rate of colon cancer patients is low (2). In China, the incidence rate of CRC was initially low; however, due to changes in lifestyle and nutritional habits, the incidence rate of CRC has increased rapidly since the 1980s (3,4). CRC now ranks as the fifth leading cause of cancer-associated mortality in China (5). Thus, several alternative therapeutic strategies are being actively pursued, including immunotherapy.

According to whether the reagent has antigen-specificity to adjust to the immunity, reagents are divided into specific and non-specific immunotherapy for cancers, of which the former is more promising due to targeting. The first step of specific immunotherapy is to identify rational antigen targets. Among the tumor antigens identified to date, the cancer-testis (CT) antigen has been recognized as one of the most potent tumor-associated antigens (TAAs). CT antigens are expressed in tumors, as well as in germ cells, but not in normal tissues. Furthermore, since the testis is an immune-privileged organ (6), cancer vaccination of CT antigens is not hypothesized to cause damage to normal tissues via autoimmune responses. NY-ESO-1 is a CT antigen that induces strong cellular and humoral immune responses (7,8). Therefore, NY-ESO-1 represents an ideal target for immunotherapeutic applications. Theoretically, patients who suffer from CRC and exhibit NY-ESO-1 expression can profit from NY-ESO-1-based immunotherapeutic strategies, as demonstrated in a previous study on vaccines targeted against NY-ESO-1 (9).

The most direct method to measure NY-ESO-1 is from tumor tissues. However, this is difficult to perform in clinical practice due to the limitations of available fresh tumor specimens. Therefore, an alternative method is required, such as testing serum samples rather than tissue samples. NY-ESO-1 has been reported to elicit humoral and cellular immune responses in patients with NY-ESO-1-positive cancers, and spontaneous serum antibodies (Abs) induced by humoral immunity against NY-ESO-1 can be detected in 40–50% of patients with NY-ESO-1-positive tumors (8,10). Furthermore, several studies have hypothesized that Ab titers against NY-ESO-1 correlate with advanced stages of antigen-positive tumors, including melanoma, transitional cell carcinoma and prostate cancer (8,11–13), indicating the later the stage, the higher the whole expression rate. Therefore, in the present study, only advanced-stage patients (stages III and IV) were selected for the analysis of serum Abs against NY-ESO-1 by ELISA. The aim of the present study was to identify patients with strong NY-ESO-1 immunogenicity to aid the selection of suitable patients for NY-ESO-1-specific immunotherapy. However, this selection process may have missed a few patients at an early stage. In addition, the dynamic expression of anti-NY-ESO-1 Abs was analyzed in 59 patients to further investigate the association with clinical status and the possible influencing factors.

## Materials and methods

### Patients and sera

In total, 155 patients were pathologically diagnosed with advanced-stage CRC (TNM stage III/IV) and hospitalized in the Department of Medical Oncology or Department of Multimodality Therapy of Oncology in the Chinese PLA General Hospital (Beijing, China) between July 2012 and December 2012. Serum samples were collected at the time of admission. There were 59 patients whose serum samples were randomly collected more than twice at different hospitalization episodes. Thus, there were 239 serum samples in total. Pathological grading was characterized according to criteria from the World Health Organization (14); tumors were classified as G1, G2 and G3 for well, moderately and poorly differentiated tumors, respectively. The study was approved by the Ethics Committee of the Chinese PLA General Hospital and written informed consent was obtained from the patients.

### ELISA

Anti-NY-ESO-1 Abs were detected by ELISA. Briefly, 1 μg/ml NY-ESO-1 purified protein (Pharos Vaccine, Inc., Seoul, Korea) or 3 μg/ml bovine serum albumin (background control; 10099141; Gibco Life Technologies, Grand Island, NY, USA) in coating buffer [15 mM Na_2_CO_3_, 30 mM NaHCO_3_ (pH 9.6)] was absorbed to flat-bottom 96-well plates (50 μl/well; eBioscience, San Diego, CA, USA) at 4°C overnight. Following blocking with 5% fetal bovine serum in phosphate-buffered saline with Tween 20 (p9416; Sigma-Aldrich; St. Louis, MO, USA) and washing, the plates were incubated for 2 h with 1:25, 1:125 and 1:625 dilutions of patient sera. Peroxidase-conjugated rabbit anti-human IgG (whole molecule; Sigma-Aldrich) was used as a secondary Ab and the reaction was allowed to proceed for 30 min. The plates were incubated with the substrate, 3,3′,5,5′-tetramethylbenzidine (860336; Sigma Aldrich), for 5–10 min and analyzed using an ELISA reader (Bio-Rad Laboratories, Hercules, CA, USA).

A strong positive reaction was defined as the optical density (OD) values exceeding the corresponding cutoff value in all the diluted serum titers (1:25, 1:125 and 1:625), while a weak positive reaction was classified as the OD values exceeding the cutoff value in two of the diluted titers. The remaining situations were defined as a negative reaction. All the experiments were performed in triplicate at different times. The cutoff value was equal to the mean OD value of the sera from the normal donors (n=10) plus three times the standard deviation. At present, there is not an ELISA kit that can be used to obtain the standard curve of NY-ESO-1; thus, this method was used to determine the reaction. Sera samples from ten normal donors served as the negative control, while a serum sample from a patient with non-small cell lung cancer (NSCLC) served as the positive control, which had been verified as NY-ESO-1 strong positive in preliminary experiments.

### Statistical analysis

Statistical analysis was performed with SPSS 18.0 software (SPSS, Inc., Chicago, IL, USA), using the χ^2^ test. P<0.05 (two-tailed) was considered to indicate a statistically significant difference.

## Results

### Baseline clinical characteristics of the patients with CRC

In total, 93 of the 155 patients with CRC were male, while 62 were female, with a mean age of 55 years (range, 18–83 years). The majority of the patients were pathologically confirmed with a diagnosis of colorectal adenocarcinoma, while there were a few cases of adenocarcinoma with mucinous adenocarcinoma and/or a signet ring cell carcinoma component and two cases of neuroendocrine carcinoma. All the patients had advanced-stage CRC, including 32 patients with stage III, 121 individuals with stage IV and two patients who were unable to be exactly grouped to stage III or stage IV. There were 83 patients whose malignancies were located in the colon, while the other 72 tumors were located in the rectum. The most frequent site of metastases was the liver, followed by the lungs and lymph nodes (Table I).

### Detection of NY-ESO-1 spontaneous Abs by ELISA

**Figure 1:**
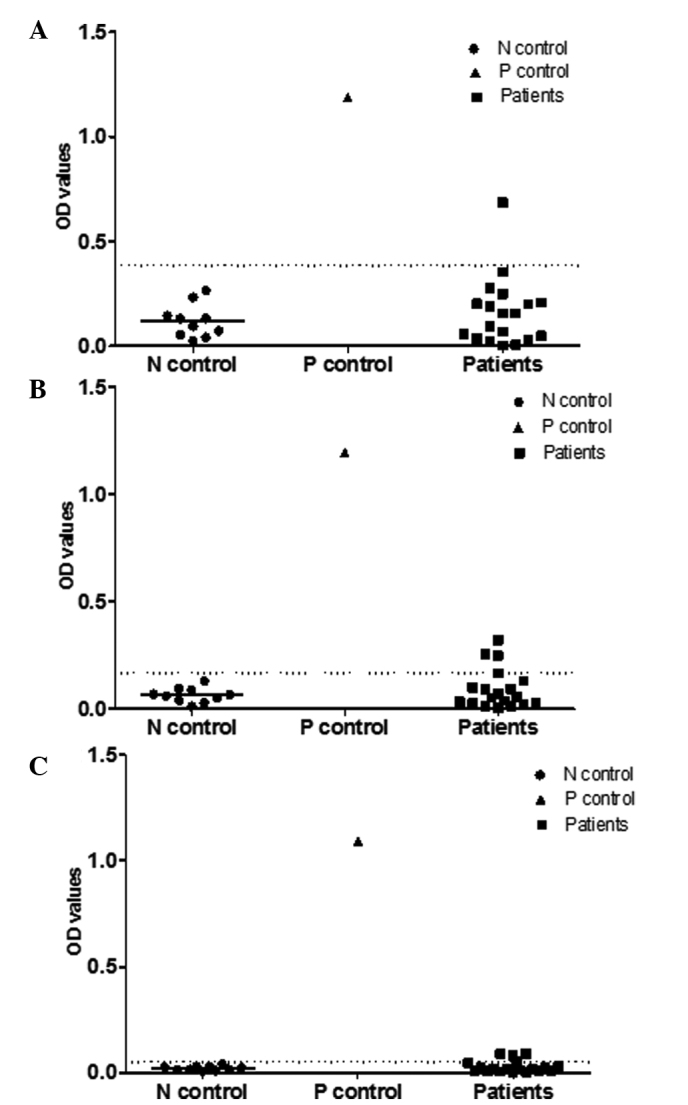
Representative ELISA results from 19 patients with colorectal cancer, with serum sample dilutions of (A) 1:25, (B) 1:125 and (C) 1:625. The dotted line represents the cutoff value (cut off value = mean OD values of the N control + 3 × standard deviation). Among the 19 patients, only one patient exhibited a ‘strong positive reaction’, where the OD values exceeded the cutoff value in the three diluted serum titers (1:25, 1:125 and 1:625). Two patients exhibited a ‘weak positive reaction’, where the OD values exceeded the cutoff values in two of the diluted titers. N control, negative control (n=10); P control, positive control (n=1; non-small cell lung cancer patient); OD, optical density.

Serum samples from 155 patients with CRC were assayed for the presence of NY-ESO-1 Abs by ELISA, using recombinant NY-ESO-1 protein as the antigen. In total, 38 of the 155 patients (24.5%) with CRC were serum Ab NY-ESO-1-positive, with 14 patients (9%) strong positive and 24 (15.5%) weak positive. In order to further explain the criteria of judging the positive/negative reactions, representative results of the ELISA from 19 patients, along with the negative control (n=10) and positive control (n=1), are provided (Fig. 1).

### Association between serum Abs and the clinicopathological parameters

No significant correlations were detected between the expression rate of NY-ESO-1 serum Abs and the clinicopathological parameters, including the age and gender of the patients, tumor location, surgical history, grading, vessel emboli/nerve invasion, local infiltration, lymph node status, metastatic status and K-ras mutation status (P>0.05). In addition, there was no statistically significant difference in the expression of anti-NY-ESO-1 Abs between stage III and IV patients due to the uneven distribution of sample size (Table II).

### Kinetic expression of serum Abs against NY-ESO-1

**Figure 2:**
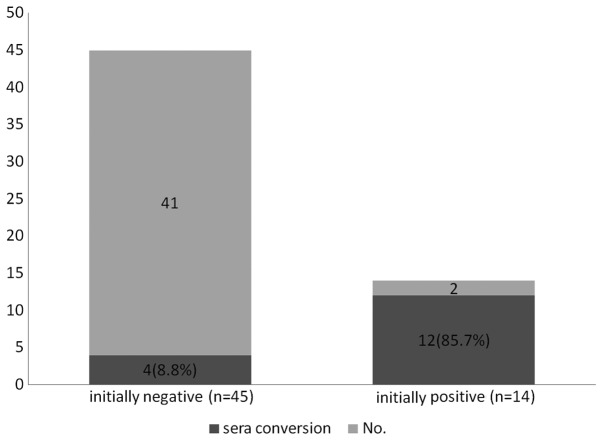
Correlation between the initial expression of NY-ESO-1 serum antibodies and sera conversion in 59 patients whose serum samples were kinetically monitored.

Serum samples were randomly collected from 59 patients with different clinical statuses (range, 2–6 times; mean, 2.4 times) in order to investigate the dynamic change in NY-ESO-1-specific Ab expression and the possible influencing factors. In total, 84 serum samples were collected. There were 14 (23.7%) NY-ESO-1-positive patients initially, and there was no statistically significant difference in the rate of NY-ESO-1-positive expression when compared with the total 155 patients (24.5%). Notably, 16/59 (27.1%) patients demonstrated NY-ESO-1 sera conversion, including mutual transformation between negative and positive or between strong positive and weak positive (Table III). In addition, among the 16 patients, 12 were initially NY-ESO-1-positive, while four patients were initially negative. This observation indicated that the initially positive patients were more likely to undergo sera conversion compared with the initially negative patients, with the incidence of 85.7 vs. 8.8% (P<0.001; Fig. 2). Similarly, sera conversion was not shown to correlate with other clinicopathological parameters, including tumor location, lymph nodes status and stage (P>0.05).

## Discussion

With the identification of a large number of TAAs, antigen-specific immunotherapy has become an important and promising therapy for cancer patients, in addition to surgery, chemotherapy and radiotherapy. Thus far, the CT antigen, NY-ESO-1, has been recognized as one of the most immunogenic TAAs, which was initially identified by serological expression cloning of a recombinant cDNA library obtained from a squamous cell carcinoma of the esophagus (10,15). The CT gene, NY-ESO-1, is widely expressed in malignant tumors of various types, including melanoma, breast cancer, esophageal cancer, gastric cancer, hepatocellular carcinomas and NSCLCs, with 10–40% of the aforementioned cancers expressing the protein (16–19), but not in normal tissues. Therefore, NY-ESO-1 has important clinical significance for antigen-specific immunotherapy. Previous studies have demonstrated that spontaneous Abs against NY-ESO-1 can be detected in a number of NY-ESO-1-positive patients with melanoma, ovarian cancer and other cancers (7,20,21). However, the expression is negative in normal individuals and NY-ESO-1-negative patients. Thus, the detection of NY-ESO-1-specific Abs using a serological method may provide the basis for NY-ESO-1-specific immunotherapy. In addition, only limited clinical data are available with regard to the expression pattern of CT genes and the associations with the pathological characteristics of CRC. Therefore, in the present study, serum Abs against NY-ESO-1 were analyzed by ELISA in 155 patients with advanced-stage CRC (stages III and IV) to investigate the clinical significance of NY-ESO-1 as a therapeutic target for CRC.

The present study detected NY-ESO-1 serum Abs in ~24.5% of patients with CRC, far higher than previous studies have reported. Li *et al* (20) reported that the frequency of NY-ESO-1 mRNA expression in CRC tissues was 9.9%, with only one serum Ab positive patient in 12 patients with NY-ESO-1-positive tumors. Of the 12 patients, 11 individuals had advanced-stage (stages III and IV) CRC. In addition, Scanlan *et al* (21) reported five autologous NY-ESO-1 Ab-positive CRC cases, in a total number of 74 patients, using a serum Ab detection array method. These differences may have been caused by a number of reasons. Firstly, the sample size in the present study is larger than in previous studies. Secondly, the present study only selected patients with stage III or IV CRC, and several studies have hypothesized that Ab titers against NY-ESO-1 correlate with advanced stages of antigen-positive tumors (8,11–13,20). Finally, serum specimens in the present study were under strict preservation and test procedures, using a serum Ab detection method with much higher sensitivity. In addition, all the samples were assayed within 14 days to ensure freshness of the samples and reduce Ab degradation. Overall, the higher expression of NY-ESO-1-specific Abs in CRC cases indicates its value as a potential target for immunotherapy. Notably, only certain patients with advanced CRC can induce spontaneous humoral immunity to NY-ESO-1, meaning that serum Ab detection misses a number of patients as compared with tissue detection. However, serum Ab detection remains a simple and quick screening method that may benefit serum Ab NY-ESO-1-positive patients.

In a previous survey of sera from normal individuals and cancer patients, Abs against NY-ESO-1 were found in ~10% of patients with melanoma, ovarian cancer and other types of cancer (7). Therefore, the expression rate of NY-ESO-1-specific Abs in patients with advanced-stage CRC is relatively high compared with other tumor types, dismissing the previous hypothesis that CRC is not an evident target for immunotherapeutic intervention due to the lack of frequently expressed tumor-specific antigens in CRC tumor tissue (20). By contrast, the results of the present study indicate that specific immunotherapy has great application prospects in CRC.

The association between NY-ESO-1 expression and prognosis remains unclear. Certain studies have indicated that NY-ESO-1 expression may be a poor prognostic factor since the presence of lymph node metastases following curative resection is one of the most important poor prognostic factors (22–24) and there is significant correlation between NY-ESO-1 expression and local lymph node metastasis (19), although the present study did not find such correlation. By contrast, due to the induction of Ab and T cell responses (7,25–27), NY-ESO-1 expression may also favor the prognosis of patients with lymph node metastasis. In the present study, the survival data were not obtained. The patients will be followed-up to further validate the correlation.

Integrated NY-ESO-1 Ab and CD8^+^ T cell responses have been reported to correlate with the clinical benefit in patients with advanced-stage melanoma treated with ipilimumab (26). However, in the present study, a correlation between the efficacy of certain chemotherapeutic agents and NY-ESO-1-specific Ab expression was not identified.

Furthermore, the present study included 59 patients of different clinical status that underwent serum collection at various time points. Kinetic monitoring of NY-ESO-1 expression demonstrated that a number of patients underwent a change in their clinical status, and notably, initial NY-ESO-1 serum Ab-positive patients were more susceptible to the sera conversion (P<0.001). However, the specific factors involved and the underlying mechanisms remain unknown. In order to investigate potential reasons, the present study analyzed the associations between clinical parameters and sera conversion; however, the results were not statistically significant (P>0.05). We hypothesize that NY-ESO-1-positive patients with spontaneous humoral immunity to NY-ESO-1 are more unstable; thus, specific immunotherapy to NY-ESO-1 may be more effective in NY-ESO-1 seropositive patients that are more susceptible to sera conversion.

In conclusion, the high expression rate of NY-ESO-1-specific Abs in CRC indicates that NY-ESO-1-based specific immunotherapy has great application potential in patients with CRC. To the best of our knowledge, previous studies have primarily concentrated on the expression of antigens in tumor tissue specimens (17–19) in order to select appropriate patients for NY-ESO-1-based antigen-specific immunotherapy. In the present study, the expression levels of serum Abs against NY-ESO-1 were analyzed in peripheral blood samples, which provided a strong basis for the clinical application of this methodology. The present study analyzed the associations between the expression of serum Abs against NY-ESO-1 and clinicopathological parameters. However, there was no statistically significant difference that required further investigation due to the uneven distribution of sample size in these variables. Finally, humoral immunity to NY-ESO-1 changed in patients with a different clinical status and the results indicated that conversion was easier in patients who were NY-ESO-1 serum Ab-positive. However, the specific underlying mechanisms require further study.

## Figures and Tables

**Table I:** Baseline clinical characteristics of the patients with colorectal cancer (n=155).

Characteristic	Patients, n (%)
Gender	
Male	93 (60.0)
Female	62 (40.0)
Pathology	
Unknown	3 (1.9)
Adenocarcinoma	123 (79.4)
Mucinous adenocarcinoma and/or signet ring cell carcinoma	7 (4.5)
Adenocarcinoma with mucinous adenocarcinoma and/or signet ring cell carcinoma component	20 (12.9)
Neuroendocrine carcinoma	2 (1.3)
Location	
Colon	83 (53.5)
Rectum	72 (46.5)
Surgical history	
None	28 (18.0)
Palliative	26 (16.8)
Radical	101 (65.2)
Stage	
Unknown	2 (1.3)
III	32 (20.6)
IV	121 (78.1)
Sites of metastases (stage IV)	
Lung	56 (36.1)
Liver	79 (50.9)
Bone	18 (11.6)
Lymph nodes	34 (21.9)
Others (adrenal gland/ovarium/peritoneal)	43 (27.7)

Median age, 55 years; age range, 18–83 years.

**Table II:** Association between the expression of NY-ESO-1 serum antibodies and clinicopathological parameters.

Parameter	Patients, n	Weak positive, n (%)	Strong positive, n (%)	Positive, n (%)	P-value^a^
Gender (n=155)					
Male	93	15 (16.1)	10 (10.8)	25 (26.9)	0.640
Female	62	9 (14.5)	4 (6.5)	13 (21.0)	
Location (n=155)					
Colon	83	11 (13.3)	7 (8.4)	18 (21.7)	0.659
Rectum	72	13 (18.1)	7 (9.7)	20 (27.8)	
Surgical history (n=155)					
None	28	4 (14.3)	1 (3.6)	5 (17.9)	0.254
Palliative	26	5 (19.2)	0 (0.0)	5 (19.2)	
Radical	101	15 (14.9)	13 (12.9)	28 (27.7)	
Grading^b^ (n=134)					
G1/G1–2	11	2 (18.2)	0 (0.0)	2 (18.2)	0.755
G2	79	10 (12.7)	9 (11.4)	19 (24.1)	
G3/G2–3	44	8 (18.2)	3 (6.8)	11 (25.0)	
Vessel emboli/nerve invasion (n=112)					
Yes	35	8 (22.9)	2 (5.7)	10 (28.6)	0.278
No	77	9 (11.7)	9 (11.7)	18 (23.4)	
Local infiltration (n=113)					
1	1	0 (0.0)	0 (0.0)	0 (0.0)	0.052
2	9	3 (33.3)	3 (33.3)	6 (66.6)	
3	10	2 (20.0)	1 (10.0)	3 (30.0)	
4	93	13 (14.0)	7 (7.5)	20 (21.5)	
Lymph node status^c^ (n=109)					
0	21	4 (19.0)	2 (9.5)	6 (28.6)	0.786
1	53	6 (11.3)	4 (7.5)	10 (18.9)	
2	35	6 (17.1)	4 (11.4)	10 (28.6)	
Metastatic status^d^ (n=153)					
0	32	3 (9.4)	3 (9.4)	6 (18.7)	0.585
1	44	8 (18.2)	6 (13.6)	14 (31.8)	
2	41	9 (22.0)	3 (7.3)	12 (29.3)	
≥3	36	4 (11.1)	2 (5.6)	6 (16.7)	
Stage (n=153)					
III	32	3 (9.4)	2 (6.3)	5 (15.6)	0.493
IV	121	21 (17.4)	12 (9.9)	33 (27.3)	
K-ras status (n=37)					
Wild type	20	1 (5)	1 (5)	2 (10)	0.159
Mutant type	17	4 (23.5)	0 (0.0)	4 (23.5)	

a Statistical analysis was performed using the χ^2^ test.

b Grading was characterized according to criteria from the World Health Organization (G1, G2 and G3 for well, moderately and poorly differentiated tumors, respectively).

c Lymph node status was classified by the seventh edition of the CRC TNM classification.

d Metastatic status was determined by the number of metastatic sites.

CRC, colorectal cancer.

**Table III:** Conversion of NY-ESO-1 serum antibodies in patients with a different clinical status (n=16).

Condition of sera conversion	Cases, n
Positive→Negative	6
Negative→Positive	2
Positive→Negative and Negative→Positive	2
Strong/Weak positive→Weak/Strong Positive	6
